# Dormancy release and germination of *Taxus yunnanensis* seeds during wet sand storage

**DOI:** 10.1038/s41598-018-21469-9

**Published:** 2018-02-16

**Authors:** Fangyuan Bian, Jianrong Su, Wande Liu, Shuaifeng Li

**Affiliations:** 1 Research Institute of Resources Insects, Chinese Academy of Forestry, Kunming, 650224 China; 2 China National Bamboo Research Center, Hangzhou, 310012 China; 3 Key Laboratory of High Efficient Processing of Bamboo of Zhejiang Province, Hangzhou, 310012 China; 4 Pu’er Forest Ecosystem Research Station, China’s State Forestry Administration, Kunming, 650224 China

## Abstract

Dormancy is an innate constraint on germination that occurs across all life forms. In this study, we investigated the seed dormancy release and germination characters of *Taxus yunnanensis* by exploring the seed morphology, permeability, germination inhibitors, endogenous hormones, and embryo germination *in vitro* during wet sand storage. Our results showed that seeds and embryos grew to a critical size to germination and permeability increased with the extension of storage. Seed coat and kernel methanol extracts reduced *Brassica campestris* seed vigor index. The *in vitro* embryo germination rate increased by 12.20% after storage for 30–360 d, whereas seed germination occurred after 450 d. Gibberellic acid and zeatin riboside contents were relatively stable, whereas abscisic acid (ABA) content decreased; indole acetic acid (IAA) content and the IAA/ABA ratio showed increasing trends. These results indicate that ABA is the key inhibitor of germination in *Taxus*. The chemical(s) in seed coat and kernel cause the inhibition of seed germination. Taken together, *Taxus* seeds have morphophysiological dormancy, in which the embryos can continue to grow and hormone imbalance inhibits further development and germination. Further, seed dormancy is active even during the middle of storage and shows “double peaks” during the entire dormancy process.

## Introduction

Seed dormancy and germination are distinct physiological processes, and the transition from dormancy to germination is not only a critical developmental step in the life cycle of plants but is also important for agricultural production^1^. Seed dormancy prevents seeds from germinating under non-favorable physical conditions^2^. In many wild species, seeds are dormant at maturity and do not germinate until dormancy release after dispersal even when subjected to optimal conditions^2,3^. Germination is a complex trait that is influenced by endogenous and environmental factors^1,4^.

The endogenous causes of seed dormancy include factors such as embryo development, seed coat (testa) impermeability, and phytohormones. Seed germination is regulated in a concerted manner that involves the generation of growth potential in the embryo to overcome the mechanical resistance of the endosperm and testa layers^5^, which is controlled by hormonal balance. The seed coat plays a critical role in the control of dormancy in many species^6^ and has been shown to regulate permeability to water, oxygen, or germination inhibitors that leach from the seed^7–9^. Phytohormones play key roles as signaling molecules for communication between the three seed compartments (embryo, endosperm and seed coat) to coordinate appropriate seed formation^10^. They can also act as important signals influencing seed development processes such as maturation, dormancy, and germination^11^. Plant endogenous hormones have important physiological effects on the process of seed formation, and the major ones are indole acetic acid (IAA), gibberellic acid (GA), zeatin riboside (ZR), and abscisic acid (ABA)^12^. These endogenous hormones play a critical role in plant growth, cell division, and elongation. Drastic changes in IAA levels have been reported to be one of the signals of embryogenesis, and bioactive GA contributes to embryo development^13^. The prominent hormone in dormancy and germination control is the germination inhibitor ABA. Generally, ABA biosynthesis and sensitivity increases during seed development and maturation to prevent premature germination^14^. Responses to hormonal signals in the endosperm and embryo influence the rate of germination.

Dormancy and germination are also affected by environmental factors^15,16^. External ecological factors such as temperature, light, humidity, and ventilation status have remarkable influence on the formation, maintenance, and elimination of dormancy. Seeds of *Taxus mairei* are known for their deep dormancy, which can only be broken by a procedure involving warm stratification, followed by cold stratification^17^. Dormancy and germination cues might enable the colonization of new locations with different seasonality by ensuring germination under appropriate seasonal conditions, thereby reducing extinction risk and providing the opportunity for subsequent adaptive divergence^18^. In production practice, *Taxus* seeds are usually placed on a sand bed blended with wet sand for storage over one year to germinate^19,20^. During this release of dormancy, the window of environmental conditions at which the seed can germinate is slowly opening^16^.

*Taxus yunnanensis*, a perish tertiary relic species in southwestern China, is an endangered species^21,22^ that is well known for its content of effective natural anticancer metabolite taxol and heteropolysaccharides. Over the past centuries, *Taxus* populations have been declining in number and size, and conspecific regeneration beneath *Taxus* canopies has almost disappeared^23–26^. Although seeds of most *Taxus* species are protected in many areas and many studies have been conducted on their cytobiology^27^, seed dispersal^28^, and spatial patterns^29^, little is known regarding the dynamic characteristics of seed dormancy and germination in *T. yunnanensis*. This study aimed to investigate the seed structural, physiological, and metabolic events taking place during dormancy and germination. A better understanding of its reproductive physiology might provide valuable information concerning the propagation of these hard-to-germinate species and facilitate complementary seed dispersal, thus enhancing population regeneration and expansion.

## Results

### Seed morphology and embryo structure

Storage time affected the embryo: the embryo length, rate, and volume increased with increasing storage time (Table 1). The long axis and transverse section of seed, embryo length, seed size, and other characteristic indexes increased gradually with storage time. Increase in storage time from 30 d (S30) to 360 d (S360) significantly increased seed long axis, seed transverse axis, seed longitudinal axis, embryo length, seed volume, and embryo volume by 3.7%, 2.8%, 6.1%, 6.1%, 12.2%, and 16.7%, respectively. Seed volume after storage for 360 d was not significantly different from that after storage for 90 d (S90), 180 d (S180), and 270 d (S270). The embryo volume of S360 was significantly greater by 27.3%, 23.5% and 16.7% than that of S90 (*P* < 0.01), S180 (*P* < 0.01), and S270 (*P* < 0.05), respectively.

**Table 1:** Seed characters of *T. yunnanensis* at different storage times.

Storage time	Seed long axis (mm)	Seed transverse (mm)	Seed longitudinal (mm)	Embryo length (mm)	Seed volume (mm^3^)	Embryo volume (mm^3^)
30 d	6.69 ± 0.22b	4.89 ± 0.20b	4.33 ± 0.14b	2.60 ± 0.14b	74.36 ± 5.90b	0.70 ± 0.12b
90 d	6.89 ± 0.33ab	4.89 ± 0.17b	4.48 ± 0.31ab	2.58 ± 0.24b	79.24 ± 9.52ab	0.66 ± 0.17b
180 d	6.94 ± 0.40a	4.94 ± 0.24ab	4.38 ± 0.29b	2.69 ± 0.28ab	79.26 ± 12.71ab	0.68 ± 0.19b
270 d	6.94 ± 0.39a	5.00 ± 0.31ab	4.41 ± 0.21b	2.66 ± 0.25ab	80.23 ± 8.81a	0.72 ± 0.16b
360 d	6.95 ± 0.39a	5.03 ± 0.25a	4.61 ± 0.38a	2.77 ± 0.27a	84.69 ± 13.00a	0.84 ± 0.24a

The letters a, b, and c indicate significant differences (*P* < 0.05) among seed tissues within each storage time according to the LSD test.

**Figure 1:**
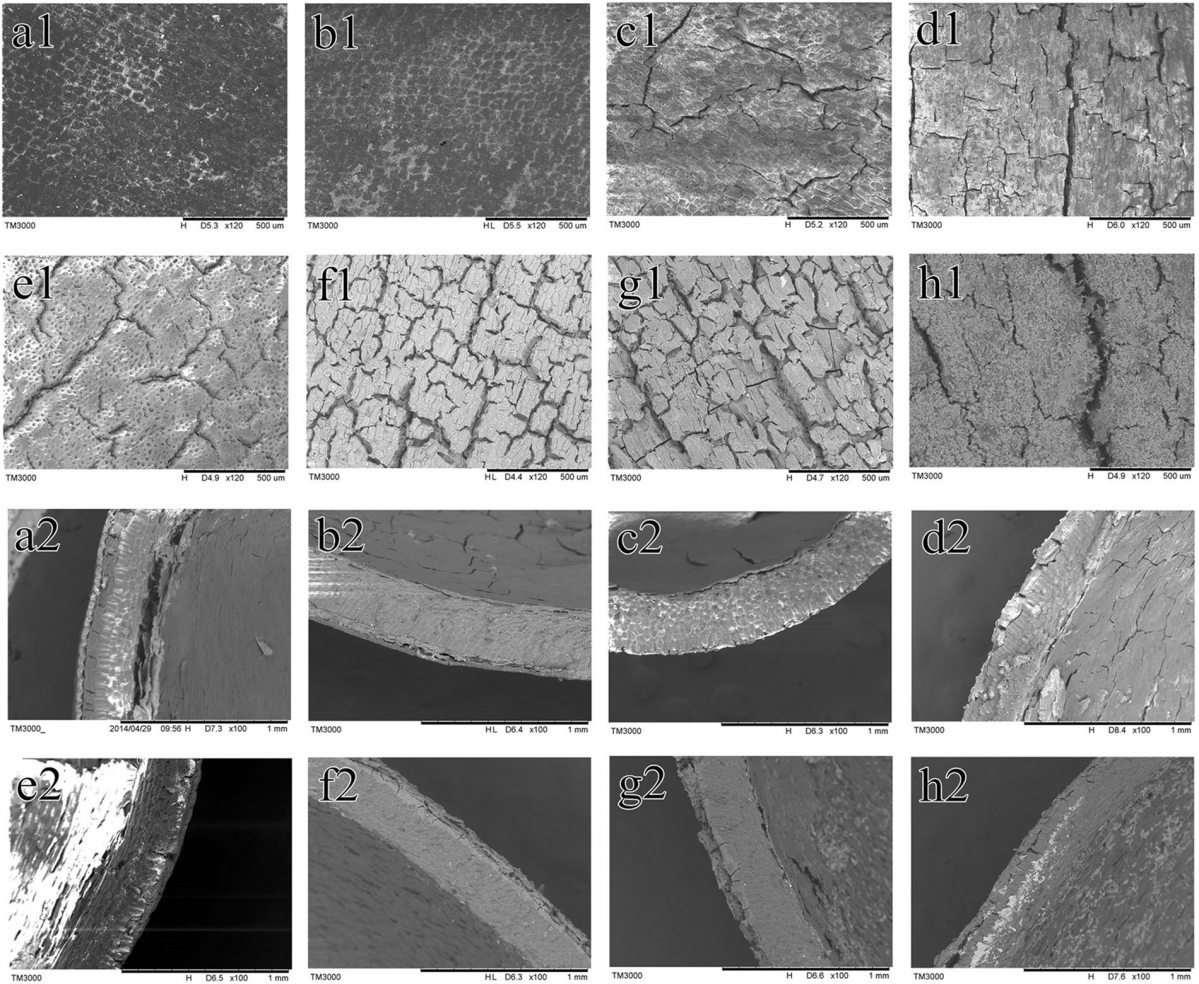
The scanning electron microscopy analysis results of the seed coat of *T. yunnanensis* at different storage times. (**a**) S90 spermoderm; (**b**) S180 spermoderm; (**c**) S270 spermoderm; (**d**) S360 spermoderm; (**e**) S90 acid-treated spermoderm; (**f**) S180 acid-treated spermoderm (**g**) S270 acid-treated spermoderm (**h**) S360 acid-treated spermoderm. 1, surface coat; 2, cross-section.

The changes in seed coat epidermis during storage of *T. yunnanensis* seeds are shown in Fig. 1; seeds acid-treated for 40 min (AT) were used as control. The seed coat was hard and consisted of 3 layers. The outer coat consisted of a layer of thick-walled cells with scales, rendering a waxy and horny appearance. The stratum corneum cells were small and closely arranged, and their cell walls were thick. The mesosperm consisted of several layers of thick-walled cells of cork having hard texture. The inner layer of the seed was membranous and consisted of several layers of stone.

The seed coats of S90 had small scattered holes, but was generally intact. The pores of S180 seed coats had begun to increase. More cracks were noted in the S270 seed coat. The S360 seed coat had larger, numerous cracks, and the surface layer was uneven and severely damaged. The seed coats of the S90 AT had sparse cracks, the stomata were arranged more clearly, and the outer coat was blurred. The S180 had a relatively dense crack. The S270 outer coat was partly removed and the number of cracks were higher. The surface seed coat of S360 AT was carbonized. A fluffy substance was noted on the seed coat, and the cracks were obvious. A large area of the seed coat was separated from the mesosperm.

### Water permeability of the seed coat

**Figure 2:**
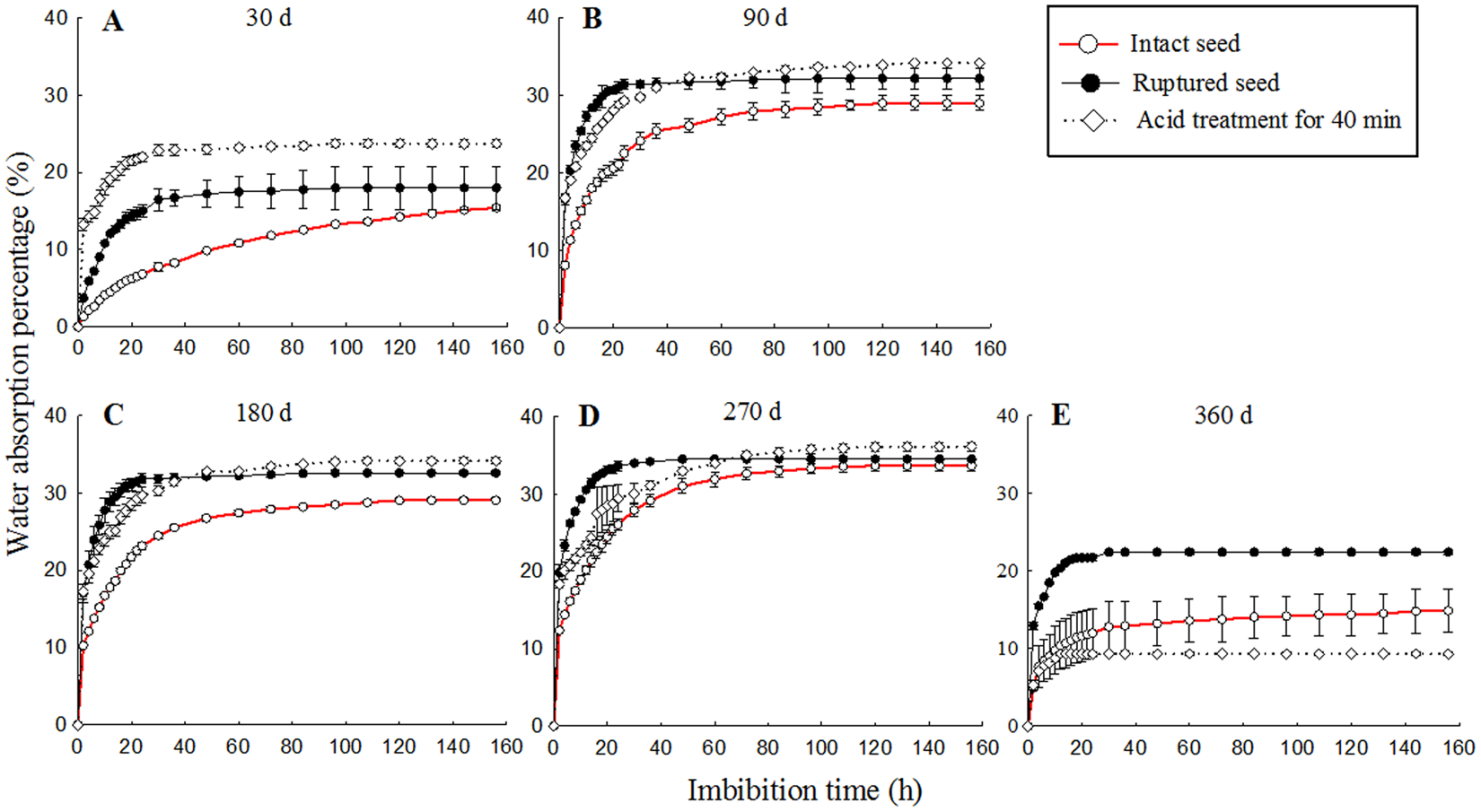
Water absorption dynamics of *T. yunnanensis* seeds subjected to different storage times. (**A**) storage for 30 d (S30); (**B**) storage for 90 d (S90); (**C**) storage for 180 d (S180); (**D**) storage for 270 d (S270); (**E**) storage for 360 d (S360).

The water permeability of the treated seeds at different storage times is shown in Fig. 2. The water content of S90 intact seeds was significantly different from that of the ruptured and AT seeds (*P* < 0.01), but the difference was not significant. The water content of S180 AT seeds was significantly higher than that of ruptured seeds (*P* < 0.01) and intact seeds (*P* < 0.001), and that of ruptured seeds was significantly higher than that of intact seeds (*P* < 0.001). The percentage of S270 AT seeds was significantly higher than that of intact seeds (*P* < 0.05), and no significant difference was noted between AT and cracked seeds. A significant difference in water absorption was noted between the S360 ruptured seeds and AT seeds (*P* < 0.01).

The water permeability test of *T. yunnanensis* seeds showed that, with the extension of storage time, the water absorption rate of seeds increased gradually, the time of stabilization became shorter, and the water permeability ability was also enhanced. The water absorption of intact seeds of S270 and S360 was similar to that of cracked seeds. The percentage of water absorbed by the S360 intact seeds was higher than that of AT seeds, probably because of the large number of cracks on the intact surface of seeds. After soaking in concentrated sulfuric acid for 40 min, the surface of AT seeds was carbonized, and concentrated sulfuric acid easily entered the seeds, leading to the inactivation of AT seeds. Thus, AT seeds remained viable for a shorter time and had weaker water absorption capacity (Fig. 2).

### *Brassica campestris* seed vigor index after methanol extract treatment

**Figure 3:**
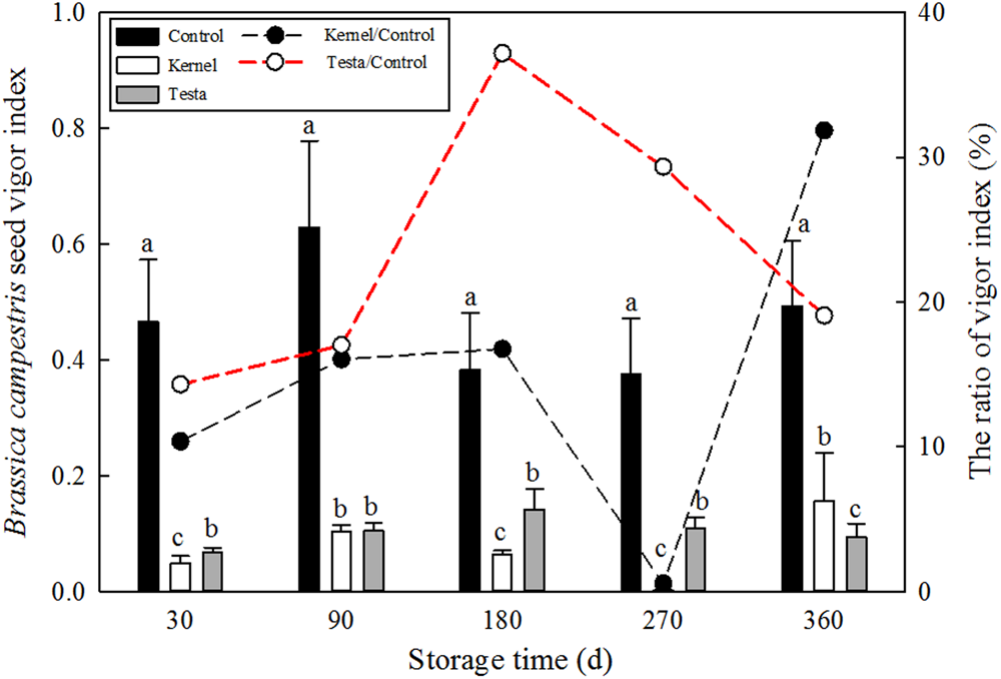
Effects of methanol extract from *T. yunnanensis* seeds on the seed vigor index of *Brassica campestris* at different storage times. The letters (a,b and c) indicate significant differences (*P* < 0.05) among seed tissues across each storage time according to the LSD test.

The effect of the methanol extract of the seed coat of *T. yunnanensis* on the simple vitality index (VI) of *Brassica campestris* seeds was in the following order per the number of days of treatment: 180 > 270 > 90 > 360 > 30 d. And the kernel of *T. yunnanensis* had significantly different among the treatments (*P* < 0.001; 360 > 90 > 180 > 30 > 270 d) (Fig. 3). The VI was significantly higher after treatment with S180 seed coat extract than that after treatment with the other extracts (*P* < 0.001), and that after treatment with S270 seed coat extract was significantly greater than those of S360 seed coat extract (*P* < 0.05), but not significantly different from that after treatment with S90 seed coat extract.

The VI of *B. campestris* was markedly different among the different storage times of control (*P* < 0.01). The average growth of *B. campestris* in control was 100% compared to the percentage of growth after treatment with *Taxus* kernel/control and seed coat/control (Fig. 3). The VI of *Taxus* seed coat was the highest (37.2%) at S180, and that of *Taxus* seed kernel was the highest (31.8%) at S360.

### Seed endogenous hormones

**Figure 4:**
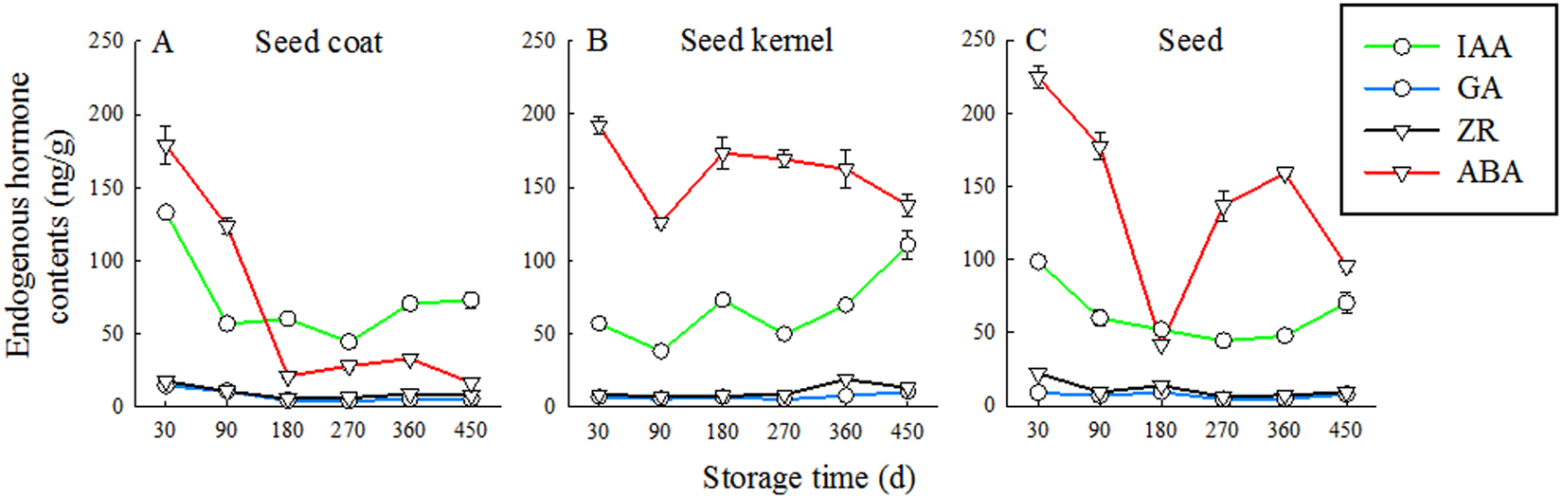
Dynamic changes in endogenous hormone content of *T. yunnanensis* seeds across the storage times.

In the seed coat, the content of IAA increased gradually after S90 and decreased slightly at S270 (Fig. 4). The ABA content rapidly decreased at first (S30–S180), and then decreased gradually (S180–S450). In the kernel, the content of GA and ZR increased slightly from S180 to S450 and remained at a low level. The content of IAA was the lowest in S90 (37.95 ng/g FW) and reached the highest value at S450 (110.49 ng/g FW), which was 2.9 times higher than that of S90. The ABA content gradually decreased and reached the minimum value (126.13 ng/g FW) in S90. In the seeds, the levels of GA and ZR varied from S30 to S450 and remained at a low level. The IAA content decreased from S30 to S270 and then increased from S270 to S450. The ABA content was the lowest in S180.

**Figure 5:**
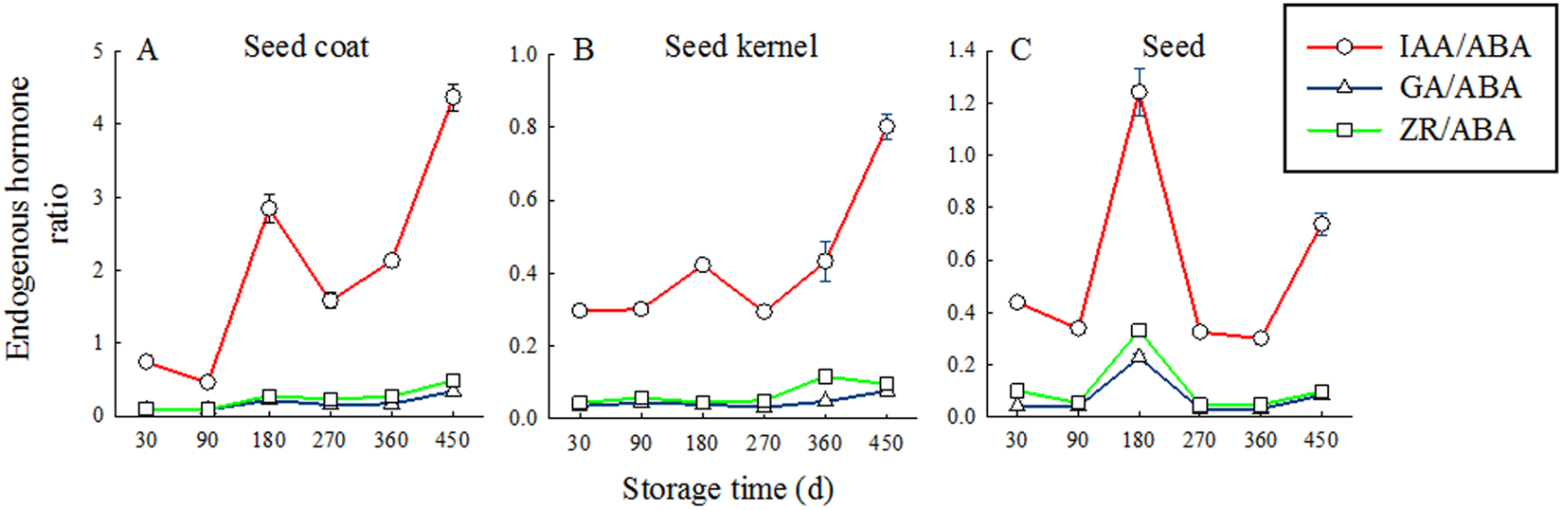
Dynamic changes in the endogenous hormone ratio of *T. yunnanensis* seeds across the storage times.

The changes in endogenous hormone ratios of *T. yunnanensis* seed at different storage times are shown in Fig. 5. The ratios showed similar dynamics for the seed coat and kernel. The contents of GA/ABA and ZR/ABA showed an upward trend at S30–S450, but at a small range. The IAA/ABA content increased sharply at S90–S180 and declined at S180–270; a rapid increase was noted at S270–S450, peaking at S450. The ratio of total IAA/ABA content in the seed coat was higher than that of seed kernel. At S180–S450, IAA content of the seed coat was 2.8, 1.6, 2.1, and 4.4 times higher than those of ABA, respectively.

In the seeds, the levels of GA/ABA and ZR/ABA remained stable from 30 to 450 d and peaked at 180 d. The trend of IAA/ABA content in seed coat, seed kernel, and seed was the same and showed a small peak at 180 d, decreased at 270 d, and then continued to increase.

### *In vitro* embryo germination rate

**Figure 6:**
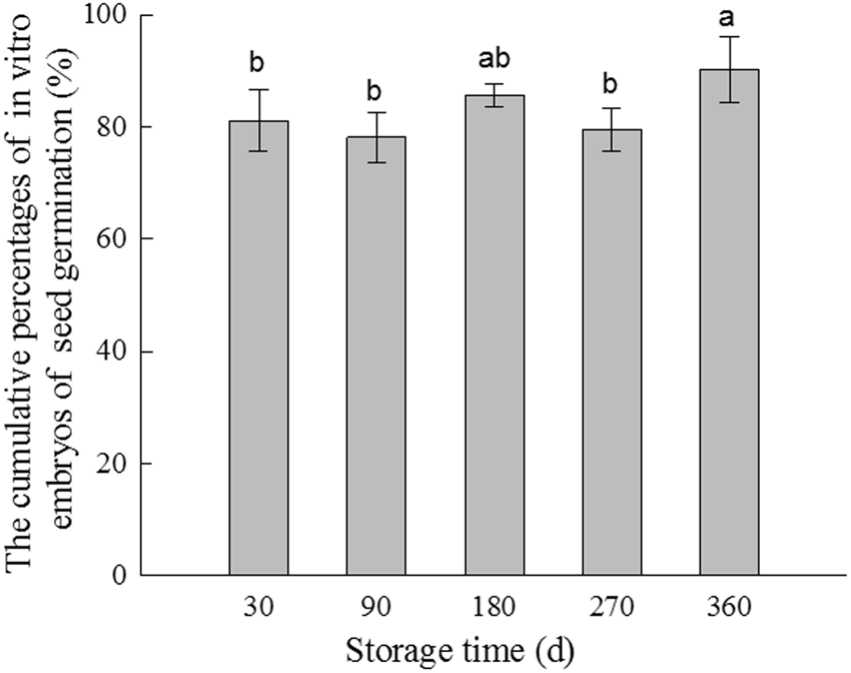
Germination rate of *in vitro* embryos obtained from seeds subjected to different storage times. The letters (a,b and c) indicate significant differences (*P* < 0.05) among seed tissues across the storage times according to the different independent samples of non-parametric Kruskal–Wallis H test. The Mann–Whitney *U* test was used to compare the two germination rates.

Significant differences were noted in the germination rates of S30–S360 (*P* < 0.05) (Fig. 6). The germination rate of S180 embryos showed a small peak; that of S270 embryos, decreased slightly; that of S360 embryos, increased; and green shoots grew out of the seed coat in S450 seeds. The S360 germination rate was the highest (90.3%), followed by that of S180 (89.9%). The germination rate of S360 and S180 showed no obvious difference, but was significantly higher than that of S360 and S270 seeds (*P* < 0.05). The *in vitro* embryo seed germination was correlated with other dormancy attributes, such as testa permeability (at 156 h), the VI ratio (mean value of kernel/control, and testa/control), and phytohormone balance (IAA/ABA). Testa permeability and the methanol extract of *T. yunnanensis* seed on the VI of *B. campestris* seeds (Seed VI) both had a clear and significant effect on germination (Table 2).

**Table 2:** Generalized Linear Model summary for *in vitro* embryo of seed germination.

Variable	Estimate	Std. Error	t value	P value
Intercept	79.807	3.963	20.138	<0.001
Testa permeability	−0.285	0.088	−3.228	<0.01
Seed VI	0.583	0.168	3.467	<0.01
Phytohormones	−1.072	2.616	−0.410	0.690

## Discussion

Seed germination is a complex process that begins with the absorption of water and ends when the radicle breaks through the seed coat^5^. An intact impermeable seed coat protects an embryo from fluctuations in humidity that could accelerate ageing, and thus might also preserve the inherent longevity of physically dormant seeds^30^. The seeds can germinate and exit the seed coat once the seed coat is disrupted and water can reach the embryo^31^. Seed permeability is closely related to seed coat structure. Researchers have identified cutin-containing layers and demonstrated their role in regulating seed permeability^32^. The cuticle is a hydrophobic film covering aerial plant structures that appears before seeds during land plant evolution. It limits transpiration and gas exchange with the environment, and protects the plants from pathogens and insects^33^. In our study, the *Taxus* seed coat surface had more and larger cracks as wet sand storage time increased. The cuticle of the testa was gradually damaged and became rough. The local cuticle dropped and began to reveal the testa; water could then more easily enter the seed. During the 156 h observation, the seeds showed a rapid growth at 20 h, which slowed down at 48 h, and finally reached a steady trend. The S360 seeds had lower water absorption capacity than that of the others. Under long-term storage in wet sand environment, the S360 seeds cracked and germinated with high water content, resulting in a lower percentage of water uptake. The seeds imbibed and absorbed water, thereby increasing respiration and metabolism and thus promoting the germination of seeds. The seed length, seed volume, embryo length, and embryo volume grew to a critical size for germination. Therefore, the physical characteristics of seed coats were related to seed dormancy.

Previous studies have shown that *Grevillea* seed coat extracts reduced seed germination and seedling growth of other species such as barley, canola, lupin, and ryegrass seeds by 48, 57, 10, and 38%, respectively^34^. In our study, germination inhibitors were also found in the seed extracts of *T. yunnanensis*, and had a significant negative effect on *B. campestris* seeds. The seed coat and kernel methanol extract of the S180 and S360 slightly inhibited the VI of *B. campestris* seeds. Moreover, the inhibitory effect of the kernel was considerably stronger than that of the seed coat at S180 and S270, which is opposite to the trend observed for S360. Therefore, during wet sand storage, the inhibitors might be transferred between the seed coat and the kernel.

In developing seeds, ABA is necessary to induce the synthesis of reserve proteins and lipids, as well as for the onset of seed dormancy and the acquisition of desiccation tolerance^35^. The profile of ABA levels observed in our study is consistent with those observed in other species such as *Taxus mairei*^17^, in which the maximum ABA levels were found at the beginning of maturation, followed by a decline. The strong seed dormancy of *T. mairei* could be caused by a high ABA content and underdevelopment of the embryos in freshly shed seeds^17^, and the lower ABA levels with seed maturation ensured the facilitation of germination^36^. ABA has been shown to play an important role in regulating embryo development and seed germination. Plant hormones have interactive effects, and hence the production of each might depend on the production of the others^37^. IAA and GA have been shown to have signaling functions during processes involved in plant growth and development, including seed germination^38^. IAA is the most important hormone for somatic embryogenesis^39,40^. The growth and development of embryos is believed to be controlled by auxin transport. IAA can influence seed germination in the presence of ABA^41^. In our study, ABA content decreased with the prolongation of storage time, whereas that of other hormones such as IAA, GA, and ZR increased. This is consistent with the findings of Yan *et al*.^12^ who showed that ABA content was significantly negatively correlated with that of the three hormones mentioned above (*P* < 0.05). Hormone content is not maintained at a constant level during dormancy. ABA is usually reduced during seed dormancy release^42^. The balanced relationships among these hormones play an important regulatory role in seed dormancy. In the middle of seed storage time, the IAA/ABA, IAA/ABA, GA/ABA, and ZR/ABA of the S180 seed coat and kernel peaked, indicating that each part of the seed was active. In the seed coat and intact seed, the endogenous hormone ratio IAA/ABA was more than 1, indicating that the levels of growth hormones were higher than those of inhibiting ones. In S360–S450, the ratios of IAA/ABA, GA/ABA, and ZR/ABA were at a high level. These results suggest that ABA is a germination inhibitor in *Taxus* seeds and hormone balance changes during dormancy.

In *Arabidopsis thaliana*, ABA signaling was associated with deeper dormancy in winter, whereas alleviation of dormancy in spring coincided with the repression of ABA signalling^43^; both hormonal and environmental cues are involved in epigenetic modifications. The ability of individual plants to change their growth and physiological responses to their environment is known as phenotypic plasticity. Seed traits such as dormancy and seed size can vary in mean value and population heterogeneity in response to environmental conditions^8^. Moisture is required for embryos to imbibe and germinate, and different species have different threshold water potentials that can support germination at different temperatures^44^. The seeds of wet sand storage need to undergo a series of changes in environmental temperature. The seeds were collected and stored in winter, and exposed to a period of low temperatures to release physiological dormancy. The embryos continued to grow, and the relatively high temperature in summer stimulated the seeds. Temperature variation during seed maturation affects primary seed dormancy by regulating coat permeability. The highest temperatures in Kunming were noted in May and June when it was S180. The S360 seeds once again underwent a low temperature stratification. The degradation of storage reserves produced solutes, including starch, proteins, and oils, which can contribute to the generation of the embryo growth potential and radicle protrusion in the embryo. Baskin and Baskin^2^ considered dormancy cycling as an adaptation that regulates the germination timing so that it occurs at the time of the year when seedlings can become established. Wet sand storage provided a breathable and moisturizing environment; meanwhile, the mixture of sand and seed could increase the friction of the seed surface, which contributed to dormancy break.

Seed dormancy is a mechanism by which seeds can inhibit their germination to wait for more favorable conditions (secondary dormancy)^45^. Seed germination is an important life-cycle transition because it determines subsequent plant survival and reproductive success. According to the dormancy classification of Baskin & Baskin^2^, which is reviewed comprehensively by Finch-Savage & Leubner-Metzger^46^, the seed dormancy type of *T. yunnanensis* is morphophysiological, in which the embryos can continue to grow and hormone imbalance inhibits further development and germination. In this study, GA and ZR contents were relatively stable in all parts of *T. yunnanensis* seeds, whereas ABA content decreased and the IAA content and IAA/ABA ratio showed rising trends; combined with the physical characteristics of seed coats and tissue extracts, this meant that S450 seeds could germinate out from the seed coat. Thus, *T. yunnanensis* seeds can be considered to have physically ripened during wet sand storage. The induction and release of dormancy are controlled by various regulators, such as testa permeability, the VI of germination inhibitor, and phytohormones. The relative strengths of these regulators are correlated and influenced by environmental factors during seed maturation and storage. The seeds of *T. yunnanensis* are even active in the middle of storage and show “double peaks”: sand storage for 180 d and sand storage for 360 to 450 d. The germination rate of the embryo *in vitro*, the biological detection of the extract, and the changes in hormone levels can support the viewpoint that there exists a “double peaks” phenomenon during the seed dormancy of *T. yunnanensis*. Our results on seed dormancy breaking and germination characters of *T. yunnanensis* might increase the understanding of the dynamics of the establishment phase of the life cycle of propagating this medicinal plant. *Taxus* seeds could provide a model for determining the end of seed development and start of germination.

## Materials and Methods

### Study Area and Species

The field experiments were conducted at Jingdong Station (23°56′–24°50ʹ N, 100°21′–101°15′ E) of the Research Institute of Resources Insect, Chinese Academy of Forestry in Yunnan Province, southwestern China. The elevation of this field station is 1200 m a.s.l. The mean annual temperature is 18.3 °C, with a range from −2 °C in January to 37 °C in July. The mean annual precipitation is 1100 mm with a seasonal distribution, primarily occurring in June to September^47^. *Taxus yunnanensis* (*Taxus wallichiana* var. *wallichiana*) is a small to medium-sized evergreen shrub or tree, distributed in patches in the undergrowth of coniferous and broadleaved mixed forests. *T. yunnanensis* was planted by hand in 2008.

### Seed samples and burial

The red aril *T. yunnanensis* seeds were collected in December 2013. Pure sand and 6 kg seeds were mixed at a ratio of 3:1 in a foam box (100 cm length, 50 cm width, 30 cm height), that contained 15 cm sand, and then a layer of fine sand and a layer of seed; the top layer of the box was covered with non-woven fabric. The sand was turned over and the right amount of distilled water was added for every 10 days, in order to maintain air permeability and moisture at 60–80%. The seed samples were stored in a shaded room having annual average temperature of 16.5 °C. The seeds were removed after storage for 30 d (S30), 90 d (S90), 180 d (S180), 270 d (S270), and 360 d (S360), respectively. They were rinsed under clean running water in a sieve (<2 mm) and dried naturally at room temperature for 24 h until observation of their physiological characteristics.

### Seed and embryo morphology

The superficial structure of the embryo was observed using a digital optical microscope (VHX-1000; Keyence Co.). Seed size was expressed as the long axis, transverse diameter, and longitudinal diameter, which were measured using an electric vernier caliper at an accuracy of 0.01 mm. Seed was measured according to its long axis, transverse diameter, and longitudinal diameter, and embryo volumes were measured using a digital optical microscope (VHX-1000; Keyence Co.) as reported by Bian *et al*.^48^. Twenty-five seeds were randomly selected by quartering, and measurements were repeated three times.

Twenty seeds of every storage time were removed, and 10 seeds were randomly selected by quartering for etching with 98% H_2_SO_4_, washed under clean running water in a sieve (<2 mm) and dried naturally. Scanning electron microscopy (TM3000; Hitachi High Technologies Co.) was performed to observe the surface and cross-sectional structure of the seed coat at 15 kV, observing the sample at 120x and 100x magnification, respectively.

### Seed moisture

This experiment included three treatments: complete seed, about one-tenth of seed coat was cut, and seeds treated with 98% H_2_SO_4_ for 40 min. Thirty complete and broken seeds each were weighted and placed in a 250 ml beaker, soaked with distilled water, and allowed to swell at room temperature. In addition, 30 acid-treated seeds were weighted and rinsed with slowly running water for 24 h, and then soaked in water. Three types of seeds were weighted every 2 h before 24 h, every 6 h after 24 h, and every 12 h after 36 h, until they reached a constant weight. Each treatment had three replicates. Seed water absorption rate was calculated as the difference in mass before and after soaking divided by the mass before soaking.

### Bioassay of seed extracts

Vigor index (VI) tests were performed on three replicates of 100 *Brassica campestris* seeds each to determine whether germination inhibitory substances existed in the extracts of *T. yunnanensis* seeds. One hundred seeds each from the S30, S90, S180, S270, and S360 experiment were randomly selected. They were separated into seed coat and kernel parts. These seed parts were separately ground. Samples were extracted with 50 ml of 80% methanol at 4 °C for 36 h; this extraction process was repeated once. The extract was distilled at 55 °C and flushed off with distilled water, which was 25 ml constant volume. *B. campestris* seeds germinated in a phytotron at temperatures of 28 °C. The final germination percentage was calculated after 72 h. Seedling lengths of 10 randomly selected seedlings were determined, and the VI was calculated. Each treatment had three replicates. The VI was determined using the following equations:

$${\rm{Vigor}}\,{\rm{index}}\,({\rm{VI}})={\rm{seedling}}\,{\rm{length}}\,({\rm{cm}})\times {\rm{final}}\,{\rm{germination}}\,{\rm{percentage}}\,( \% )$$

### Determination of plant hormones

All seeds of S30, S90, S180, S270, and S360 were immediately frozen in liquid nitrogen after their fresh weight had been determined, and then stored at −80 °C until analysis. Samples were extracted with 10 ml 80% (v/v) methanol containing 1 mmol/L butylated hydroxytoluene in an ice-cooled mortar to prevent oxidation. The extract was incubated at 4 °C for 4 h and centrifuged for 15 min at the same temperature. The supernatant was passed through a C18 Sep-Pak cartridge (Waters, Milford, MA, USA). The efflux was collected and dried in N_2_. The residues were then dissolved in 0.01 mol/L phosphate buffer solution (pH 7.4), and the concentrations of IAA, GA, ZR, and ABA were determined using ELISA. Microtitration plates (Nunc, Roskilde, Denmark) were coated with synthetic IAA, GA, ZR or ABA–ovalbumin conjugates in 50 mmol/L NaHCO_3_ buffer solution (pH 9.6) and stored overnight at 37 °C. Ovalbumin solution (10 mg/ml) was added to each well of the plates for blocking nonspecific binding. After the plates were incubated for 30 min at 37 °C, standard IAA, GA, ZR, ABA, samples, and antibodies were added and incubated for an additional 45 min at 37 °C. The antibodies against IAA, GA, ZR, or ABA were produced according to the method described by Weiler *et al*.^49^. Horseradish peroxidase-labeled goat anti-rabbit immunoglobulin was then added to each well and incubated for 1 h at 37 °C. After the incubation, orthophenylenediamino substrate solution was added to each well. The plates were then kept in the dark at 37 °C for substrate reaction. After 15 min, 3 mol/L H_2_SO_4_ was added to each well to terminate the substrate reaction. The absorbance of each well was measured at 490 nm by using the ELISA Recorder (Model DG-3022 A; Huadong Electron Tube Factory, Shanghai, China). In this study, the percentage recovery of each hormone was calculated by adding known quantities of standard hormone to a split extract. All percentage recoveries were >90%, and all sample extract dilution curves paralleled the standard curves, indicating the absence of nonspecific inhibitors in the extracts.

### Embryo germination experiment

Before this experiment, the seed coat of S30, S90, S180, S270, and S360 seeds were removed using a scalpel. Thirty-six kernels were sterilized with 70% ethanol for 1 min, 0.1% mercuric chloride for 6–7 min, followed by rinsing in sterile water 4 times. Endopleura and endosperm were excised with a sterile scalpel and needle under aseptic conditions, and embryos with 1/2 endosperm were inoculated on Murashige-Skoog (MS) medium. Somatic embryos were transferred using a sterile tweezer onto MS hormone-free medium with 3% sucrose and 0.8% agar at pH 5.8. These embryos were maintained on the same medium. All cultures were incubated in a chamber at 25 ± 1 °C with 8 h dark and 16 h light regime of 2000 lx. Each experiment was repeated three times. Germination date was recorded for *in vitro* embryos of each seed storage.

### Data analyses

The data were analyzed using SPSS 19.0 (SPSS Inc.). Means were compared using one-way analysis of variance. Multiple comparisons were used to determine the differences in seed water absorption ability with different storage times by using the least significant difference (LSD). The *in vitro* embryo germination rate from seeds was compared across independent samples by using non-parametric Kruskal–Wallis H test. Further, Mann–Whitney *U* test was used to compare the two germination rates. The cumulative percentages of *in vitro* embryo seed germination were plotted using the R code and analyzed using Generalized Linear Models (GLM) to assess the effects of testa permeability (at 156 h), the VI ratio (mean value of kernel/control and testa/control), and phytohormones (IAA/ABA). The GLM was used to consider not only the primary correlations between attributes but also the overlap patterns among these correlations.
